# Mediating alcohol use in Eastern Nigeria: a qualitative study exploring the role of popular media in young people's recreational drinking

**DOI:** 10.1093/her/cyx043

**Published:** 2017-05-07

**Authors:** Emeka W. Dumbili, Lesley Henderson

**Affiliations:** 1 Department of Social Sciences, Media and Communications, College of Business, Arts and Social Sciences, Brunel University London, Kingston Lane, Uxbridge, London, UK; 2 IInstitute of Environment, Health and Societies, Brunel University London, Kingston Lane, Uxbridge, London, UK

## Abstract

Nigeria has high levels of alcohol consumption, and little or no regulation of the alcohol industry. There is a dearth of studies exploring young adults’ drinking in a Nigerian context with only a few predominantly quantitative surveys. These do not explore the social meanings attached to drinking practices nor do they shed light on potential gender differences and how these are mediated by popular media. This qualitative study addresses this gap with semi-structured interviews involving 31 undergraduate students. It identifies that media consumption shapes drinking behaviour in ways which are highly patterned and gendered. Participants with high consumption of both Hollywood films and popular American reality television series associate heavy alcohol consumption with high social status, economic independence and gender equality. By contrast, Nollywood (local) films which are intended to act as moral tales and warn of the dangers of drinking appear paradoxically to support participants’ views of alcohol as positive (alleviating anxiety, depression and menstrual discomfort). Nigeria currently has no serious regulation of alcohol on television which is embedded in everyday life. Attempts to develop wider public health campaigns and policies should take this saturated media landscape into account to develop harm reduction strategies which are linked directly to media literacy programmes.

## Introduction

Mass media portrayals of alcohol are pervasive, and their impact on adolescents and young adults are significant [1, 2]. The media, especially television, is not only young people’s source of knowledge of alcohol, but also an agent of socialization into their drinking [3–5].

According to Miller [6], ‘audiences are the opium of television—it craves them, longing to control their time and space… Television has physical existence, a history of material production and consumption, in addition to its renown as a site for meaning making’. Numerous studies identify that young people’s exposure to onscreen alcohol portrayals through music videos [7, 8], advertisements [9–11] and films is prevalent [12, 13], and that this influences their drinking.

Alcohol consumption in entertainment television, especially drinking by principal characters in films engenders young people’s intention to use alcohol and their onset drinking [3, 14]. It also has been found to influence the consumption of larger quantities among those who already consume alcohol [4, 15]. A key reason is that attractive media characters have considerable appeal for young people, serving ‘as ‘super peers’… *and* providing models and information about alcohol use that may not be available in peer group or family’ [16].

Research conducted in Europe suggests that alcohol portrayals in films facilitated onset drinking amongst low-risk (i.e. those who are unlikely to drink) German adolescents [17], engendered heavy episodic drinking among Scottish youths [18] and binge drinking among young people who already consume alcohol in Germany, Italy, Poland, the Netherlands, Iceland and Scotland [19]. Similarly, a study of middle school adolescents in Argentina and Mexico highlighted that alcohol consumption in movies increased their likelihood of alcohol use, current alcohol consumption and exacerbated binge drinking [20]. Of significant interest is that, out of the 873 films Mejia *et al.* [20] analysed, as many as 780 were Hollywood movies.

Hollywood movies (and other American television series) frequently contain scenes of alcohol on-screen [21], most of which reinforce alcohol consumption positively [3, 22]. Again, exposure to Hollywood movies has been found to encourage alcohol initiation among American youth [23] and to exacerbate alcohol-related harms [24]. In this light, Hollywood industry is seen as a disease vector that begins in the USA where they are produced and spread to other countries ‘where Hollywood movies are a staple in the media diets of children, adolescents’ and young adults [20].

Although a large body of literature has examined young people’s exposure to media portrayals of alcohol and its effect on their drinking, as shown above, they are largely quantitative studies, and mostly American or Eurocentric. Little is known about how media mediate substance use in Africa and even less is known about Nigeria. The dearth of research in the Nigerian context is serious because Nollywood (Nigerian movie industry) is not only ranked the third largest movie industry worldwide [25, 26], it also produces the largest number of films per month (between 200 and 250 films [27]).

Additionally, psychoactive substances are common in Nollywood films [26]. For example, out of 479 local films Aina and Olorunshola [26] examined, 268 portrayed at least one scene of alcohol, cannabis, tobacco, cocaine or heroin use. Importantly, 197 out of these 268 films depicted alcohol consumption, making it the most-used substance [26]. Therefore, it is possible that those who are exposed to these alcohol depictions may be influenced to consume alcohol. Although alcohol consumption among young people is culturally taboo in Nigeria [28], young people in contemporary Nigeria do drink, with many using heavy drinking to ‘perform gender’ (i.e. to construct/express a range of masculine/feminine identities) [29]. There is also evidence of diverse drinking patterns and motives among students in Nigeria [30, 31] and alcohol-related problems such as accidents, mental disorder, anxiety, amongst others, are increasing [32–34] but to date, there is no empirical evidence on how the media may be part of these trajectories.

Indeed, qualitative research conducted in Western countries identifies how drinking to excess is normalized amongst youth in friendship networks [35–37] and how students in particular employ heavy drinking to construct a range of gender identities [38]. Studies also highlight the importance of exploring the meaning of drinking as a rite of passage and some of the cultural differences in the perceptions of men and women who can ‘handle large amounts of alcohol’ [39, 40]. The use of alcohol amongst people in friendship groups to construct gender identities is consistent with gender theorists’ assertion that gender is performed in collaboration with others [41, 42]. For example, West and Zimmerman [41] posit that ‘a person’s gender is not simply an aspect of what one is, but, more fundamentally, it is something that one *does*, and does recurrently, in interaction with others’.

Because gender is accomplished in the presence of onlookers (who judge gendered behaviour as ‘appropriate’ or otherwise), those who construct gender identities engage with different resources to accomplish expected gendered behaviours, and one important resource is alcohol [38, 43]. Being that gendered behaviour is learned and internalised through the processes of gender socialization [41], the media occupies a powerful position in the learning process, especially contemporary Western media that often portray how alcohol is associated with gender identity constructions, successes and wealth [7]. Therefore, with the growing globalisation of Hollywood movies and the popularity of American/Western culture amongst young Nigerians [44], it could be argued that audiences in Nigeria live within a saturated media landscape (foreign and local) that may affect their drinking behaviour. This study, therefore, aims to explore young people’s awareness and engagement with alcohol portrayals in the media and their perception of how media mediate their drinking behaviours.

## Materials and methods

It was important to explore the social context of drinking amongst young people who lived on and around University campus as these locations are key sites for excessive alcohol consumption in Nigeria [30]. This study was conducted on a university located in a city of Anambra State, south-eastern Nigeria between September and December 2013. Semi-structured interviews were conducted with 22 male and 9 female students (aged 19–23 years). All but one of the participants was from the Igbo (The Igbo ethnic group is one of the three major ethnic groups in Nigeria. They live in the eastern part of the country where the data for this study were collected.) ethnic group. After receiving ethical approval from the Nigerian University and UK’s University Ethics Board, participants were recruited across nine faculties on the university campus using word-of-mouth and snowballing techniques [45]. These methods were required for the successful recruitment of female participants. While alcohol consumption amongst young people elicits sociocultural disapproval in Nigeria, young female drinkers are particularly stigmatised [29, 46]. Written informed consent for participation was sought from the participants. All participants’ names have been replaced with pseudonyms.

After piloting the protocol, an interview guide that consisted of 12 main questions (with additional probes) was developed. Key questions include: do you watch television? About how long do you spend watching television in a day/week? What type of film/movie do you watch? Why do you prefer this movie genre? Do you see alcohol portrayal in the movies you watch? Can you share with me how alcohol is portrayed in the movies you had seen? Participants were also asked to self-report how much alcohol they consumed on each drinking occasion. Because there is no Government recommended safe drinking guide in Nigeria, in that there are no alcohol policies [47], they were asked to describe their drinking in terms of the number of bottles they consume. Thirty-one semi-structured interviews lasting 33–90 min were conducted. All of the interviews were conducted in the English language as the participants were fluent English speakers. The interviews were recorded in full with the permission of the participants.

## Data analysis

The interviews were transcribed verbatim and a thematic analysis was undertaken [48]. Thematic analysis was adopted not only for its flexibility and easy-to-follow steps, but for the fact that we aimed to generate ‘unanticipated insights’ on how young people make sense of their social lives with alcohol through the combination of theory-driven and data-driven analysis [48, 49]. Indeed, as Braun and Clarke note, thematic analysis is useful ‘when investigating an under-researched area, or you are working with participants whose views on the topic are not known’ [48]. As Silverman [50] suggested, a preliminary analysis was initiated immediately after the first interview. Tentative coding schemes were developed manually at an early stage after the first six interviews [48] with initial extracts categorised into broad themes and subthemes. Collaborative analysis between the authors and another senior academic was also adopted at this stage to ensure analytical rigour [51]. This provided an early grasp of the data [52], and some of these tentative themes, which were grouped manually, became the parent nodes while others were condensed [53] into different child nodes that formed the thematic coding framework when the data were imported into the NVivo software.

When all of the 31 interviews had been transcribed, the transcripts were read several times and cross-checked and reconciled with the audio recordings before importing them into the NVivo software. Following this, a number of queries were conducted, the first of which was a word frequency query to gain an insight into the words most frequently used by the participants and how this could help in understanding the patterns within the whole data set. It also helped with further coding the data [54].

When the coding was completed, the nodes were read thoroughly to identify incompatible quotes. Through these means, such quotes were condensed or expanded into the existing child nodes or new nodes were created [48] before running matrix coding queries. At the end of the matrix queries, the nodes were exported to a word document and read several times. Here, some comparisons with the few tentative themes that had been generated manually were made before recording the patterns of meaning [48] from the key themes that had been identified.

## Results

### Media consumption amongst the participants

Our analysis found evidence of diverse media use patterns among the participants. Here we wish to explore how television is consumed specifically and how this might relate to perceptions of influence on drinking behaviours. The key themes/subthemes informing our findings pertain to the ways in which alcohol is portrayed in positive light in Hollywood films as against negative portrayals in local films. Specifically, we focus on three main themes: normalization of drinking in Hollywood, aspirational drinking in Hollywood and moral tales of drinking in Nollywood. While some of the participants watched television every weekday, others mainly watched films and other programmes on weekends. Relatedly, half of our male participants watched television during daytime while the majority of the females reported watching television during night-time. Our analysis also shows that the nature of television watching related to length of time spent viewing. Thus, those who watch television during weekdays do not spend more than two hours on a viewing occasion, whereas weekend viewers tended to watch for four or more hours. Those participants who watched television during the night (weekdays or weekends), especially female participants spend longer in their viewing as compared with daytime viewers.

Importantly, we found that while media use pattern is gendered, the type of movies that participants watched is gendered too. All the male participants watched movies and none indicated that he watched popular reality television series. All the female participants also watch movies and reality television series. The data revealed that while nearly all of the male participants watch ‘action movies’, none of the female participants reported viewing this movie genre. The female participants rather stated their preference for ‘epic’ or ‘drama’ movies. These gendered issues of taste and preferences confirm findings from previous studies [55]. Interestingly both male and female participants watch High School films (i.e. popular American feature films with romantic themes, where the main action is set within high schools). Our data revealed that nearly all the male participants watch only Hollywood films and six of the females combine Nollywood and Hollywood movies. The context of media consumption is important because as we explore in the following section, the type and origin of movie, and the participants’ viewing pattern, to a large extent, determined their awareness of how alcohol is represented and their perceptions of its influence on their own drinking.

### Hollywood normalises drinking

Participants were invited to share their views on the type of movies they watch, the reasons for their preference, whether or not they typically see onscreen alcohol portrayals in the movies and their perceptions of how alcohol was represented. Many of the male participants indicated that they watch Hollywood movies, and all reported seeing alcohol depicted. When they were asked to explain how it was depicted, their accounts show that alcohol appeared to be normalised within different genres such as drama, epic and High School films as evidenced in Boniface’s and Peter’s words: ‘it’s kind of rare where you watch American films, and you don’t see drinking in that film’; ‘I just noticed that they enjoy it, and ladies drink alcohol too…They enjoy drinking’. The majority of the male participants demonstrated awareness of alcohol portrayals, showing how it is used in the performance of masculinity:

Movies give the impression that for you to be recognized as a strong guy you need to be a ‘good alcoholic’. In any party, there must be beer and the girls drink beer like boys, as in freely and also in large quantities just to get drunk … (Larry, Male, 21years)

A key part of the men’s accounts highlighted the way in which some of them were able to give vivid descriptions of the Hollywood films they had seen that portrayed alcohol with positive repercussions:

…There was a movie that I watched last week … where a guy met a girl, who was a very influential lady in society … They went to the same bar coincidentally. The guy and the girl got drunk, and somehow they met [had sexual intercourse]. So they had a baby, and somehow they just had to marry, and that was how the guy’s life changed, and he became influential. (Chike, Male, 21years)

Interestingly, their views on alcohol in Hollywood films were shared by all female participants whose views not only highlighted the positive representation of alcohol but also showed how characters drink freely in contrast with the reality of alcohol consumption in Nigeria where young people’s drinking would be considered taboo. In the excerpts below, female participants explain the role they imagine that alcohol plays in the everyday life of ‘typical’ Americans and how alcohol representation in Hollywood differs from portrayals in locally made movies:

In all these movies I watch, I noticed that these outsiders [foreigners] take red wine all the time. Like even if they are just eating… lunch or dinner, you’ll see them pouring themselves a glass of wine, and they are always drinking … (Chichi, Female, 23years)

In Nigerian films, alcohol use among young people is kind of restricted … I see Nigeria as more of a religious kind of country than Americans because most ‘‘Janded’’ [foreign] movies I watch, they tend to drink alcohol, especially the New Yorkers and those New Jersey people… The youth there drink alcohol …; most of their scenes are always in clubs … (Patience, Female, 23years)

The participants were not only aware of the positive images of alcohol in Hollywood films, but also reality television shows such as Jersey Shore and the America’s Next Top Model (started May 20, 2003; first aired in United Paramount Network). Both male and female participants demonstrated their perceptions of how alcohol consumption among young people is normalised in American television and movies, unlike those produced by, and about Nigeria.

### Hollywood-induced aspirational drinking

The participants were not only aware of alcohol being represented in a positive light in Hollywood movies, but importantly, the onscreen depictions were believed to have either influenced their own drinking behaviour (and their peers) or have potential to facilitate their alcohol consumption. For example:

…There is this drama that I saw; the guy does this funny thing of always having a bottle or a can of beer in his pocket … and he always drinks. Sometimes he opens the bottle with the head of his belt or he hits it on his head to open the bottle before drinking …; you really want to be like him. The guy is always the happening guy [popular] … I think I would like to go to a bar one day so that I can feel like this guy … (Chike, Male, 21years)

Chike said he watches television for up to 21 hours a week and typically drinks four bottles of beer or three bottles of stout (with 5.1% and 7.5% alcohol by volume—ABV, respectively) on each drinking occasion. One significant aspect of his account is his plan to go to a bar to practise what he saw in the movie. Another is his reference to ‘the guy is always the happening guy’. Nearly all of the male participants demonstrated their perception of how drinking without showing signs of intoxication is related to ‘fame’ [38] and showed how heavy consumption is used to construct superior masculinity [56]. Thus, Larry refers to ‘the impression that for you to be recognized as a ‘strong guy’ you need to be a good alcoholic’. Our analysis shows that consuming Hollywood films connects with men’s extant beliefs that heavy drinking or the ability to ‘hold one’s drink’ is a marker of superior masculinity [57], suggesting that ‘bodies are the site for performing masculinity’ [58].

Male participants not only reveal how positive alcohol portrayals can engender aspirational drinking, but they demonstrated their awareness of how it can also influence brand preference:

…One thing about Nigerians is that they really like portraying [imitating] what they are not… Seeing a celebrity drinking Heineken in a movie … maybe he drank it in a very unique style, you’ll now want to keep that same position when you drink. You just want to be like him. Therefore, you make Heineken your brand. Like if this ‘big man’ can really like Heineken, why don’t I try Heineken? … So you’ll go to a bar and say, ‘please give me a bottle of Heineken,’ because you just want to have those feelings that you’re like him. (Peter, Male, 23years, spends 21hours weekly watching TV, drinks beer and spirit)

Of particular interest, is his reference to the ‘Heineken brand’. In Nigeria, any product that is imported from Western countries is regarded as superior to its local counterparts. For example, despite the fact that ‘Nigerian Breweries Plc’ (owned by Heineken) localised the production of Heineken beer since the early 2000 s, many Nigerians still mistake the brand as a foreign product. In this vein, some of the male participants mentioned that Heineken brand is a beer for the ‘upper class’, and also indicated that they drink Heineken whenever they want to show off their ‘class’ to their peers in public drinking spaces. Cranwell and colleagues [7] note that music videos that portray alcohol as being associated with wealth and success do encourage over consumption. Similarly, our data show how the onscreen portrayals of this brand in foreign movies appear to authenticate and even strengthen our participants’ beliefs, to the extent that it engenders aspirational drinking.

The data also revealed that Hollywood’s High School films are popular on this campus and offer a particularly aspirational view of drinking amongst students:

… High School movies are just about fun, and their fun cannot be without alcohol … The majority of the movies we watch here are for fun and usually with beer…, so you’ll want to be like them … . It influences us … because we want to have fun like the whites. In their movies, they drink alcohol so much, so [we think] let’s do the same thing and have fun too. (Larry, Male, 21years)

Larry is among the participants who preferred Hollywood films, and he spends up to 42 hours weekly watching television. Within our sample, he engaged in drinking that was heavier than many participants and it appears that this heavy television viewing normalises his consumption of large quantities of alcohol. Additionally, all the male participants revealed that they had taken part in drinking games, and some described learning about these from ‘movies high school movies’. For example:

For those games they do at parties, I think they learned it from scenes in High School movies. I mean ‘the first to drink and drop the bottle’… (Levin, Male, 21years).

The positive portrayal of alcohol in Hollywood movies is also perceived to have influenced our female participants. For example, some believe that onscreen drinking by movie characters could lead to alcohol initiation while others have learned how to play drinking games from Hollywood films:

I’ve seen movies where friends do ‘drinking competitions’; they’ll give you a glass of wine and you’ve got to gulp it down all at once. If you take it twice, you lose the game… Even if it’s a bottle, you have to drink it without removing the bottle from your mouth until the drink finishes… Sometimes they will give drinks that have higher alcohol content, and if you vomit you lose the game. So … it’s [learned] in movies. That is where we see different competitions, and that’s where I saw for the first time that you could drink alcohol without removing the bottle from your mouth … (Chimanda, Female, 22 years)

The women’s accounts also revealed nuanced, sophisticated aspirational drinking which appears to have been influenced by their viewing of Hollywood movies and other American reality television programmes:

A character said in one American movie … that red wine is good for the heart because it helps pump blood to the heart. I have not really asked a doctor, but since then, I just started taking it… (Chichi, Female, 23years)

Interviewer: So are you saying that you started to drink red wine because you watched the movie?

Yeah it’s more like ‘professional’ because these people [movie characters] always use it for ‘professional things’… they are the ones that actually made me develop a liking for red wine. Because … if they go to meetings, or they are celebrating something, they will say, ‘this calls for wine’, and you’ll see them just open wine and pour it, and they take it with wine glasses. So it looks very ‘professional’ and ‘mature’. So that’s why I just like it. (Chichi, Female, 23years)

Female participants described in detail how not only their drink of choice but their comportment during drinking are influenced by imitating movie characters who were considered to be sophisticated and professional. Chichi consistently uses the terms ‘professional’ and ‘mature’, and highlights her admiration of Hollywood movie characters, stating that they often comported themselves in parties, conferences or business meetings by queuing up or sitting down to be served by waiters, who pour wine into their glasses. In her words, ‘*as they are talking, they sip, and they drop [put down the glass], so it’s very clean and classic*’. Her perception of these actors shows that their onscreen behaviour is quite different from what she regularly sees in Nigeria, where poor queuing culture or the act of drinking directly from the bottle prevails. She also references what she considers to be ‘sophisticated’ drinking in popular reality franchise:

Another thing that stimulated me was the ‘American Next Top Model’. So one of the tests these models had to pass was wine testing … just to show you how these people value wine. They are blindfolded, and then they bring two different types of wines … and tell you to taste this one. After taking it, you’ll taste the other one. After testing them, they will be like, can you differentiate these two wines or can you name the one that is French or an American wine? …And then you’ll see some of the models getting it right. At least … even though they don’t get both of them, they will get one right just because … wine is something they are used to … (Chichi, Female, 23years)

As a self-reported Hollywood film addict, who spends over 50 h weekly watching television, it appears that the consumption of American films may have a significant influence on Chichi’s drinking behaviour and she consumes at least two bottles of red wine per drinking occasion. The media may have also influenced her perception of social reality, in terms of social inequality and women’s subordination in Nigeria. During the interview session, Chichi argued that women should be free to consume large quantities of alcohol of their choice like men do if they wish. Other participants in the study supported the view that seeing alcohol represented in a positive light in a movie could engender students’ drinking. Both male and female participants stated that many youths identify with Hollywood movie characters, seeing them as ‘significant others’. Thus, they often copy their drinking (and non-drinking) behaviours.

Nigeria is a patriarchal society where alcohol consumption is gendered. Although the culture taboos young people’s drinking, as indicated earlier, young female drinkers are particularly stigmatised [29]. Both our male and female participants highlighted the fact that seeing their contemporaries, especially women drink freely in Hollywood films created a desire to emulate them. Our analysis reveals another way in which foreign media consumption and the students’ environment heightened this for female participants, who learned to associate drinking with women’s independence:

The [unequal drinking] orientation is changing… The media are displaying alcohol and this time around, it is no longer restricted [to males]… When you come to an environment like this university…, you meet people, you mix with friends, and you hear this orientation of equality between male and female genders; so everybody wants to try out new things. ‘Nobody wants to be left behind just because you are tagged a woman’. (Pretty, Female, 20years)

Our data reveal that while the men who view Hollywood films consume four or more bottles of beer/stout on a drinking occasion, the majority of their female counterparts also use large quantities of wine or other flavoured alcoholic beverages, highlighting the globalization of the culture of intoxication among youths [35, 39]. Together, these accounts have shed light on the participants’ perceptions of how Hollywood films may influence young people’s drinking behaviours.

### Moral tales of drinking in Nollywood

Participants also revealed that they had high awareness of alcohol portrayal in Nollywood films. In contrast with the positive portrayals they see in Hollywood movies, they perceived Nigerian films as mainly depicting drinking in a negative light:

…When the movie is centred on maybe campus life or is about youths, the intake of alcohol will be great. Let me give an example. There is a movie I saw few days back; the title is ‘‘Campus on Fire.’’ It has to do with campus cultism… In each episode, there are like six or seven guys on a table gulping down alcohol. As in, they will be drinking irresponsibly. That’s what they are trying to portray… (Genny, Female, 23 years)

While how alcohol was portrayed in a negative light was revealed, the participants also indicated that Nollywood movies often focus on the consequences of consuming alcohol:

Most of the time in Nigerian movies you see them go out to a club and they will drink to stupor, and it will make the people do some nasty things that they are not meant to do… Maybe in a family setting, the wife or the husband will be angry, and the man leaves for a bar. At the end of the day, he gets drunk and engages in some things that are not beneficial like fighting. (Chimanda, Female, 22 years)

The representation of alcohol in a negative light in Nollywood movies was also highlighted by other participants, but one unique aspect of the men’s account shows the way in which they were able to compare and contrast the different themes associated with alcohol consumption in Hollywood versus Nollywood movies:

In Nigerian movies, alcohol is taken when a guy is going through some hard times; when you want to do something bad… these are the only times you’ll see someone taking alcohol. In foreign movies, you’ll just see someone drinking with friends and laughing. They drink at parties, have fun… after that, they go to bed and sleep. The next morning they wake up and talk about what happened last night… In Nigerian movies, someone will take alcohol, then come home and beat his wife and do all sorts of bad things. (Levin, Male, 21 years)

Like Levin stated, other male participants, who indicated their preference for Hollywood movies reported that the reasons why they do not watch Nigerian films are because of their poor production values and the way in which they often over-represent the negative impact of drinking.

Interestingly, the participants also noted that Nollywood films show how alcohol consumption is used to construct superior masculinity or femininity among young people:

In the movies, when they want to be ‘bad girls’ or ‘bad boys’, definitely they will be taking alcohol. They will be showing them taking alcohol and forming [displaying that]: we are the ‘big girls’ or the ‘big boys’. (Chioma, Female, 21 years)

The data also revealed some nuances on the way in which Nigerian movies portray how alcohol consumption can be used to ameliorate anxiety and depression. For example, male participants described characters in Nollywood movies using heavy drinking in a bid to cure depression. Although this is clearly a negative portrayal, our male participants indicated that these portrayals are powerful and could possibly influence behaviour, particularly for young people facing relationship difficulties:

…Under normal circumstances, if somebody breaks your heart you’ll think over it and get rid of it, but if you are watching those videos where they normally create the impression that when people are depressed or heartbroken, they will prefer to drink, you will do that…. Even the women maybe her boyfriend annoys her so she will go to the bar and start drinking. That is the scenario they create… When you are depressed, the next thing you’ll do is go to the bar and start drinking. (Kelly, Male, 21 years)

Kelly revealed how he turned to alcohol to help his depression and how he learnt this practice through the media:

… It’s from the [local] media. I have heard a lot of people talk about that. Initially I didn’t believe the whole thing, but when I started drinking I discovered that it worked for me…, so now I have formed the habit of taking alcohol when I am depressed. (Kelly, Male, 21 years)

Kelly views local television for over 50 h weekly, and this appears to have influenced his drinking practices, in that he consumes three bottles of beer per drinking occasion, in addition to the large quantities of locally-made ‘palm wine’ (palm tree’s sap).

Among our female participants, it was found that local media influenced their brand preference with positive attributes being associated with certain brands. Ada, for instance, recalled that local television influenced her preference for ‘Guinness stout’ which she found to be particularly helpful during menstruation because: ‘‘it cleanses out the rubbish in our stomachs… if you have it with milk, it gives blood, and it also gives energy’’. Many other participants also revealed that this practice is popular among Nigerian women who use stout to ameliorate menstrual pain and perceived it as useful to enhance energy or regain blood (this is attributable to how Guinness stout is portrayed in local television [59]). When she was asked whether or not she thinks that media can influence her or her peers, Ada laughed and replied emphatically that: ‘everyone wants to do what they see on television’.

These accounts have shown that although the Nigerian media depict alcohol mainly in a negative light, some of the participants were nonetheless influenced to consume alcohol in diverse ways. While the men learnt to use the number of bottles an individual can drink to determine how manly he is, and to ameliorate depression, they also learned to associate specific brands with affluence and worldly status. The women also learnt to associate drinking with a higher social status and to ameliorate period pain.

## Discussion

The findings of this study highlight how young people’s drinking in Nigeria is mediated by media representations and aspirational ideas fuelled not only by Western images but also by local media depictions of alcohol. Our analysis found evidence of alcohol images depicted in a positive light in Hollywood movies, reinforcing previous research [20, 21] which shows that young people are exposed to the prevalent positive representations of alcohol in American films.

The lived experience of the participants showed that their viewing was gendered [55]. It also highlighted that they were aware of the positive images of alcohol in Hollywood films and demonstrated the gendered nature of the influence these images had on their drinking. While the men may have learned to associate heavy drinking with fame and superior masculinity [38, 56], their brand preference and the association of foreign brands with a higher socioeconomic capital were influenced too.

Interestingly, the fetishization of foreign alcoholic beverages evident here tends to reflect practices which refer back to Nigeria’s colonial history. During the colonization of Nigeria (1914–1960) by the British Colonial Government, imported alcoholic beverages were reified by the British Officials, who consumed foreign beverages and distanced themselves from the locally-made brands [60]. A few Nigerian elites also had access to ‘high-class European spirits’ [61]. Consequently, this became a status conferral, to the extent that, these elites associated themselves with ‘the prestige of white man’ because of consuming imported alcoholic beverages [62].

This was reinforced even when beer importation began to grow. Again, drinking British, Danish and German beers became ‘an emblem of European lifestyles and values’ [63]. The association of imported and/or industry-made alcoholic beverages with a high social status was carried over to the post-independence by the ‘westernised Nigerians’, who also disassociated themselves from the locally made beverages, describing them as the ‘choice for the poor’ [64]. Therefore, as our analysis shows, viewing Hollywood movies, especially the depictions of particular brands that are accorded foreign or expensive status in Nigeria, appears to reinforce these inherent beliefs. The positive depiction of alcohol also affected ways in which male and female participants were drinking (i.e. drinking games involving swift drinking, directly from the bottle in binge sessions).

Our findings also demonstrated sophisticated aspirational drinking among the participants, especially the women, to the extent that they not only copied the brand and admired the drinking postures of Hollywood movie characters, but they used their drinking to perform non-traditional femininity [65]. The act of identifying and admiring movie characters, to the extent of imitating their drinking style corroborates Atkinson *et al.*'s [12] assertion that young people see media characters’ drinking behaviours ‘as a guide to what are ‘normal’ drinking practices’.

The women’s perception of drinking as a marker of female independence was also mediated by the American media, but this appears to be a deviation from the traditional femininity that is anchored in the Nigerian culture, which discriminates against women’s drinking [29, 46]. Indeed, these findings demonstrate the power of American media to mediate young people’s behaviour [20, 23] and reinforce Stoolmiller *et al.*'s [66] assertion that: ‘like influenza, images in Hollywood movies begin in one region of the world then spread globally, where they may affect drinking behaviours…everywhere they are distributed’.

Our data also identified participants’ perception of the negative representations of alcohol in Nollywood films, and how male participants in particular, described this as an exaggeration of social reality. Paradoxically, the negative depiction was also perceived to influence drinking with male participants described learning to drink to enhance their low mood from these supposed moral tales. This indicates that audiences have a complex and often ambivalent relationship with media that can be difficult to predict. Our data supports other research that shows how participants may reject or subvert health related messages in popular media [67].

In this article, we explore how drinking is embedded in the everyday culture of a group of young Nigerians at University. It is a small study and relied on the accuracy of self-reported accounts of how our participants consume alcohol and local/foreign media. However, it represents a rare qualitative account of how media and alcohol are part of University life for our participants. Although, this may also be the case for most students in other countries, a focus on the Nigerian context shows a particular aspirational outlook which we argue could be based on a specific colonial past. Our research suggests that local and imported media may be important conduits of messages concerning drinking– moral tales from local media can be easily discounted in light of more aspirational messages from US media. At present, there is no regulation of alcohol via policies in Nigeria [47] and imported foreign media is seemingly unregulated too.

However, we argue that it is vital that media literacy education is aimed at facilitating the critical engagement with ‘media messages that promote risky behaviors’ [68]. Because popular media is a vital conduit of health-related messages [69, 70], media literacy can play a role in future public health initiatives in Nigeria and help to unpack the assumptions and dispel the misconceptions which we discuss here. Alcohol media literacy interventions [1] could focus on emphasising that ‘media messages are constructed’; thus, they shape rather than reflect social reality; that media messages are created with the intention to make gains and/or exert power over audiences, and that they are embedded with ‘values and points of view’ [68]. This will not only empower youths to recognise whether or not media messages they are exposed to are value-laden, biased, stereotypical and mythical, but will reduce the effect of media modelling. If the accounts of our participants are accurate, then living in a saturated ‘mediascape’ mediates both young males’ and females’ drinking behaviours in contemporary Nigeria.
